# O5.1. CLOZAPINE AND LONG-TERM MORTALITY RISK IN PATIENTS WITH SCHIZOPHRENIA: PRELIMINARY RESULTS FROM A META-ANALYSIS

**DOI:** 10.1093/schbul/sby015.215

**Published:** 2018-04-01

**Authors:** Jentien Vermeulen, Geeske van Rooijen, Marita van de Kerkhof, Arjen Sutterland, Christoph Correll, Lieuwe de Haan

**Affiliations:** 1 Academic Medical Center, University of Amsterdam; 2 Zucker Hillside Hospital, Hofstra North Shore LIJ School of Medicine

## Abstract

**Background:**

Patients with schizophrenia have a high mortality risk. The role of clozapine in the long-term mortality risk is insufficiently known. The objectives of the current study were to determine in i) all-cause long-term mortality rates and ii) specific-cause mortality rates and ratios in patients with schizophrenia with and without clozapine treatment.

**Methods:**

We systematically searched EMBASE, MEDLINE and PsycINFO and included studies that used a long-term follow-up design (i.e., ≥52 weeks) and reported on mortality in adults diagnosed with schizophrenia-spectrum disorders receiving clozapine treatment.

**Results:**

Altogether, 23 studies fulfilled our criteria, reporting on 1,166 deaths during 203,231 patient years for patients treated with clozapine. Pooling five cohort studies that included sufficient sample sizes and length of follow-up, we found an unadjusted mortality rate of 7.34 per 1,000 patient years (95%CI=4.39–10.28). Long-term, crude mortality rate ratios were significantly lower in patients treated with clozapine compared to patients without clozapine treatment (mortality rate ratio=0.59, 95%CI=0.43–0.81, p-value<0.001) as well as compared to other antipsychotic medications (mortality rate ratio=0.61, 95%CI=0.45–0.84, p-value=0.002). We found incomplete and inconsistent reporting of specific-cause mortality rates. Statistical heterogeneity was high in all analyses.

**Discussion:**

Future studies with substantial length of follow-up and uniform reporting of confounders are needed to validate these findings of a significantly lower mortality risk in patients using clozapine, in particular for the risk of cardiovascular mortality.

